# There is No Link Between Birth Weight and Developmental Dysplasia of the Hip

**DOI:** 10.1007/s43465-021-00465-8

**Published:** 2021-08-30

**Authors:** Charlotte Hanratty, Balamurugan Thyagarajan, Nicholas M. Clarke, Alexander Aarvold

**Affiliations:** 1 University of Southampton, Southampton, UK; 2 Princess Anne Maternity Hospital, University Hospital Southampton, Southampton, UK; 3 Department of Paediatric Orthopaedic Surgery, Southampton Children’s Hospital, University Hospitals Southampton NHS Foundation Trust, University of Southampton, Tremona Raod, Southampton, SO16 6YD UK

**Keywords:** Developmental Hip Dysplasia, Dislocated, Reduced but dislocatable, Reduced but dysplastic, Packaging disorder, Birth weight

## Abstract

**Aims:**

Developmental Dysplasia of the Hip (DDH) has been linked to high birth weight and packaging disorders, though the evidence is limited. This has implications on screening strategies. The aim of this study was to establish whether birth weight was truly associated with the incidence of DDH.

**Patients and Methods:**

This cohort study analysed the birth weights of all babies born at our institution over a 24 month period, between 01/01/2017 and 01/01/2019. Babies with DDH and those without DDH were compared. Babies were excluded if born before 38 weeks, had incomplete data or were a non-singleton pregnancy. Sub-analysis was performed for DDH severity (dysplastic versus subluxed/dislocated hips), breech presentation, gestational age, gender and ethnicity. Statistical analysis was performed using SPSS.

**Results:**

There were 10,113 babies born at our institution during the selected timeframe, of which 884 were excluded for prematurity, 336 for being non-singleton and 19 for incomplete data. This left 8874 for analysis, of which 95 babies had confirmed DDH. Both the Non-DDH and DDH data sets had normal distribution (Shapiro-Wilkes, *p* = 0.308 and 0.629, respectively), with mean birth weights of 3477.7 g with DDH and 3492.8 g without DDH. No difference in birth weight was found (Independent *T* test, *p* = 0.789). Females had a lower birth weight than males (3293.1 g versus 3416.6 g (*p* < 0.001)) yet have a higher incidence of DDH (ratio 6:1 in this dataset). No significant difference was found between birth weights of females with and without DDH (*p* = 0.068), nor between males with and without DDH (*p* = 0.513). There were no significant differences in birth weights even when only displaced hips were analysed (*p* = 0.543), nor according to breech presentation (*p* = 0.8). Longer gestation babies weighed more (*p* < 0.00001), yet showed no increase in DDH incidence (*p* = 0.64).

**Conclusion:**

This study discredits the belief that DDH may be related to higher birth weight, thus casting doubt on the link to DDH being a packaging problem in utero. This, therefore, allows future research to prioritise the investigation of alternative aetiologies.

## Introduction

Developmental Dysplasia of the Hip (DDH) is a congenital developmental abnormality which if untreated, causes significant pain and long-term disability [1]. Screening methods remain varied and with a little evidence base. Incidence may be as high as 30–40/1000 live births [2], however, with no gold standard test this remains controversial.

It is clear that early diagnosis is critical for DDH, as treatment in a brace is extremely successful and safe when commenced within the first few months of life [3]. This underpins the rationale for screening. However, selection of which hips to screen, and when, requires an understanding of aetiology and / or risk factors [4, 5].

The aetiology of DDH appears to be highly complex, thus identification of ‘risk factors’ for screening is challenging. As there is undoubtedly a familial link, multiple studies have investigated genetic causes, though none have found a definitive answer [6–8]. Some literature theorises that DDH is a packaging disorder, caused by a reduction in intrauterine space [5, 9, 10]. This can result in limb abnormalities at birth such as torticollis, calcaenovalgus foot, metatarsus adductus, congenital dislocation of the knee and hip dislocation [11–17]. These may present together, indicative of reduced intrauterine space being linked to the deformities [5].

Normal birth weight is defined as 2500–3999 g. Greater than 4000 g is defined as macrosomia, causing cramping in utero with restricted foetal movements [18]. Low birthweight is defined as 2499 g or less [19], and may be associated with an increased risk of hip osteoarthritis [20, 21], potentially due to lower bone mineral density (BMD) resulting from the growth restriction in utero [19]. Literature on DDH is more suggestive of a link to higher birth weight [22–25], yet males tend to have a higher birth weight than females (nationally in the UK this is 3436 g versus 3316 g, respectively) [26] whilst having a much lower incidence of DDH [27].

Historical literature highlights why high birth weight is sometimes considered a risk factor for DDH. Some of this literature suggests a link to first-borns and to oligohydramnios [14, 27, 28], while other studies attribute it to intra-uterine crowding [29–31]. These papers all present a mechanism whereby a higher birth weight would induce a higher incidence of DDH. However, there is little in the literature to support this. Where a link has been found, it is to macrosomic babies i.e. > 4000 g and > 4500g [15]. However birth weights of this magnitude are uncommon, and the numbers involved and statistical significance of these findings is unclear.

What is clear is that there remains uncertainty around the relationship between birth weight and DDH, confounding the inclusion criteria for DDH screening and aetiology. This study aims to investigate whether DDH is linked to birth weight, either high or low, through a comparison of population and DDH databases.

## Methods

### Data Source and Participants

Following approval from the Ethics and Research Governance Online (ERGO) committee (Ref. 49398), anonymised patient data was obtained from two separate sources at our institution, a large NHS teaching hospital. The data set of all births between 01/01/2017 and 01/01/2019 was accessed from the Newborn and Infant Physical Examination (NIPE) SMART database. The full dataset included Sex, Gestational Age, Birth Order, Birth Weight, mode of presentation (breech versus vertex), NHS Number, Ethnicity and Place of Birth.

All patients treated at our institution for DDH, either by Pavlik harness (if detected early) or by surgery (if detected late or if harness treatment failed), were cross referenced against the cohort of patients born at our institution within the defined time frame. Using their NHS number, these patients were extracted from the dataset of all live births, thus creating two groups—those with and those without DDH, born within the same timeframe and within the same region.

Patients were excluded if they were non-singleton pregnancy (due to multiple births usually being lighter), born outside of a pre-defined Gestational Age window of 38–42 weeks, or incomplete data. Patients treated at our institution for DDH, but born out of the region, were excluded to ensure there was one defined population being studied. Sub-analysis was performed for DDH severity (classified as Graf 2b, c or d signifying reduced/dysplastic hips, and Graf 3 or 4, signifying displaced/dislocated hips), birth presentation, gestational age, gender and ethnicity. Statistical analysis was performed using Microsoft Office Excel, the Data Analysis ToolPack and IBM SPSS Statistics.

A sample size calculation was performed using the Cochran formula to find the minimum sample size appropriate for the DDH cohort. The desired confidence level was set at 95%, the precision at 5% and the estimated proportion of the population with the attribute at 3.5% [2], as per current literature. The minimum sample size required was found to be 52.

## Results

**Fig. 1:**
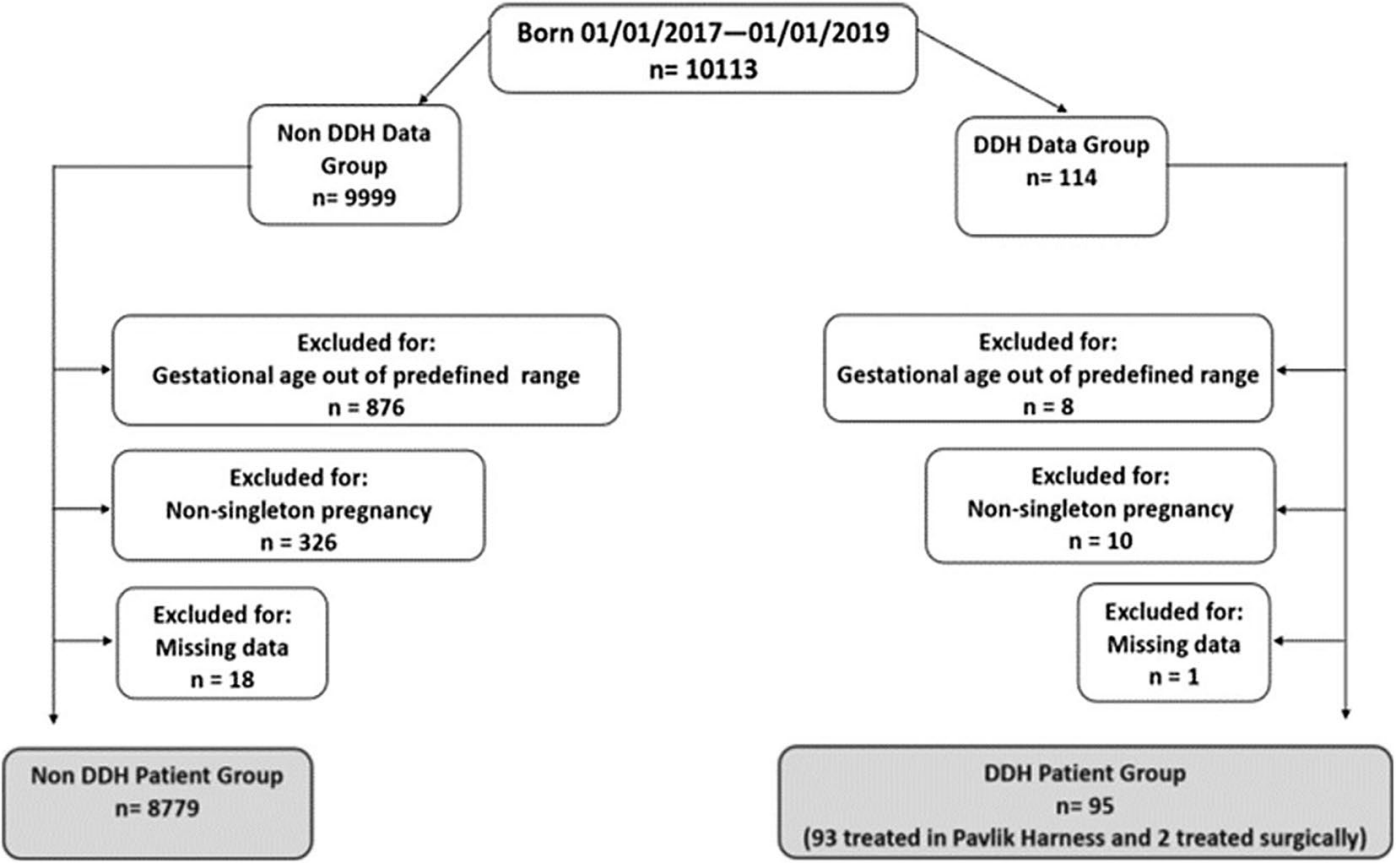
Overview flowchart detailing all patient exclusions during the data collection period

There were 10,113 live births at our institution in the 24 month period of the study. Once all exclusion criteria were applied (Fig. 1), 8874 patients remained for analysis; 95 with DDH (43 participants more than the minimum required sample size), 8779 without DDH. Of the DDH data set, 93 infants were treated in Pavlik harness, (having been detected early through the national selective ultrasound screening programme) and two were detected after walking age (i.e. had a normal peri-natal examination and had no risk factors for DDH, so did not qualify for hip USS). These two were included in the DDH data set because even though their DDH was diagnosed out with the identified timeframe, their birth date fell within the study timeframe and they were treated for DDH. The process of patient selection is represented in Fig. 1 as a flow chart.

**Fig. 2:**
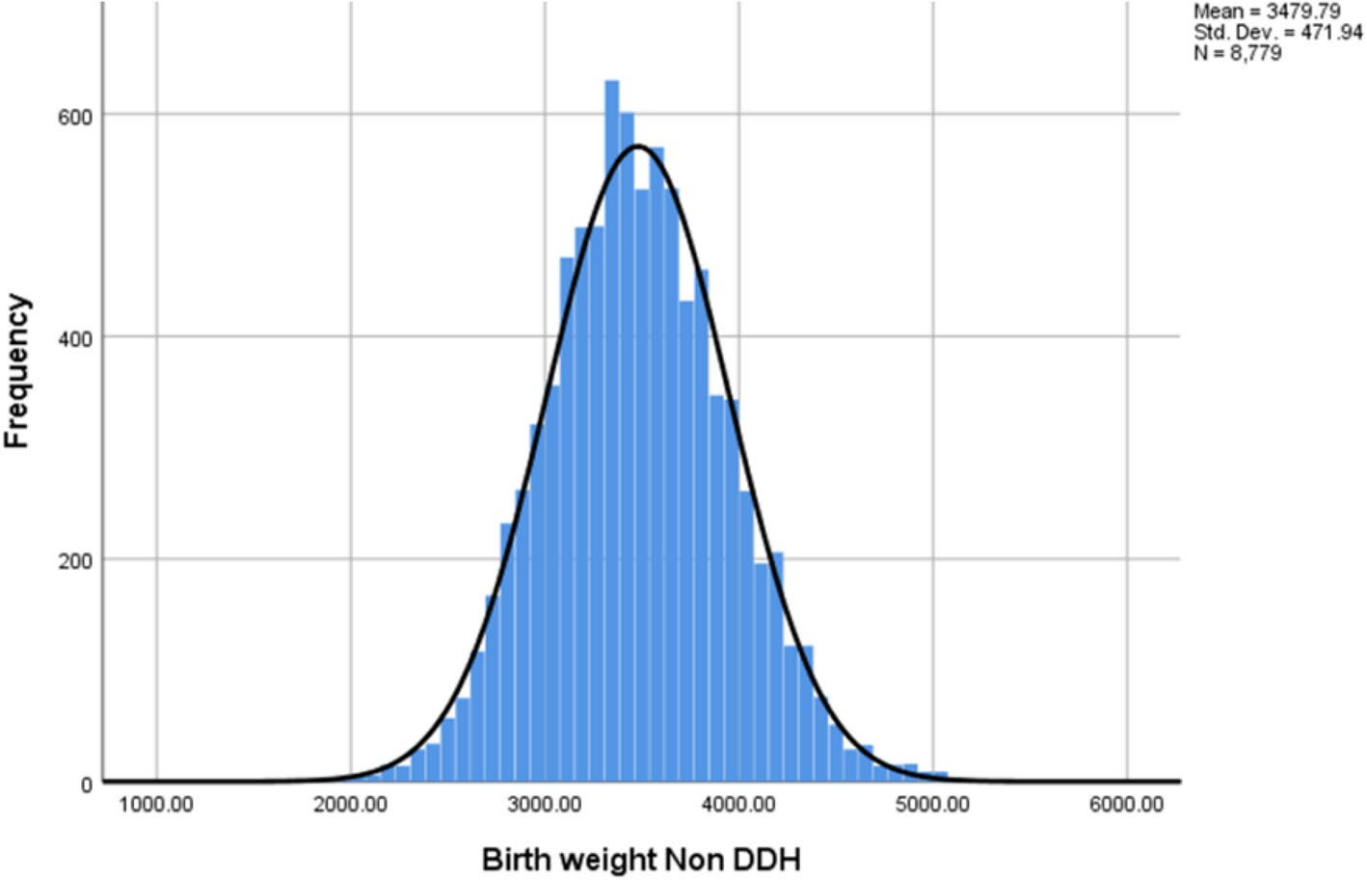
Histogram showing Non-DDH birth weights frequency, with a normal distribution line overlaid. It shows a normal skew

**Fig. 3:**
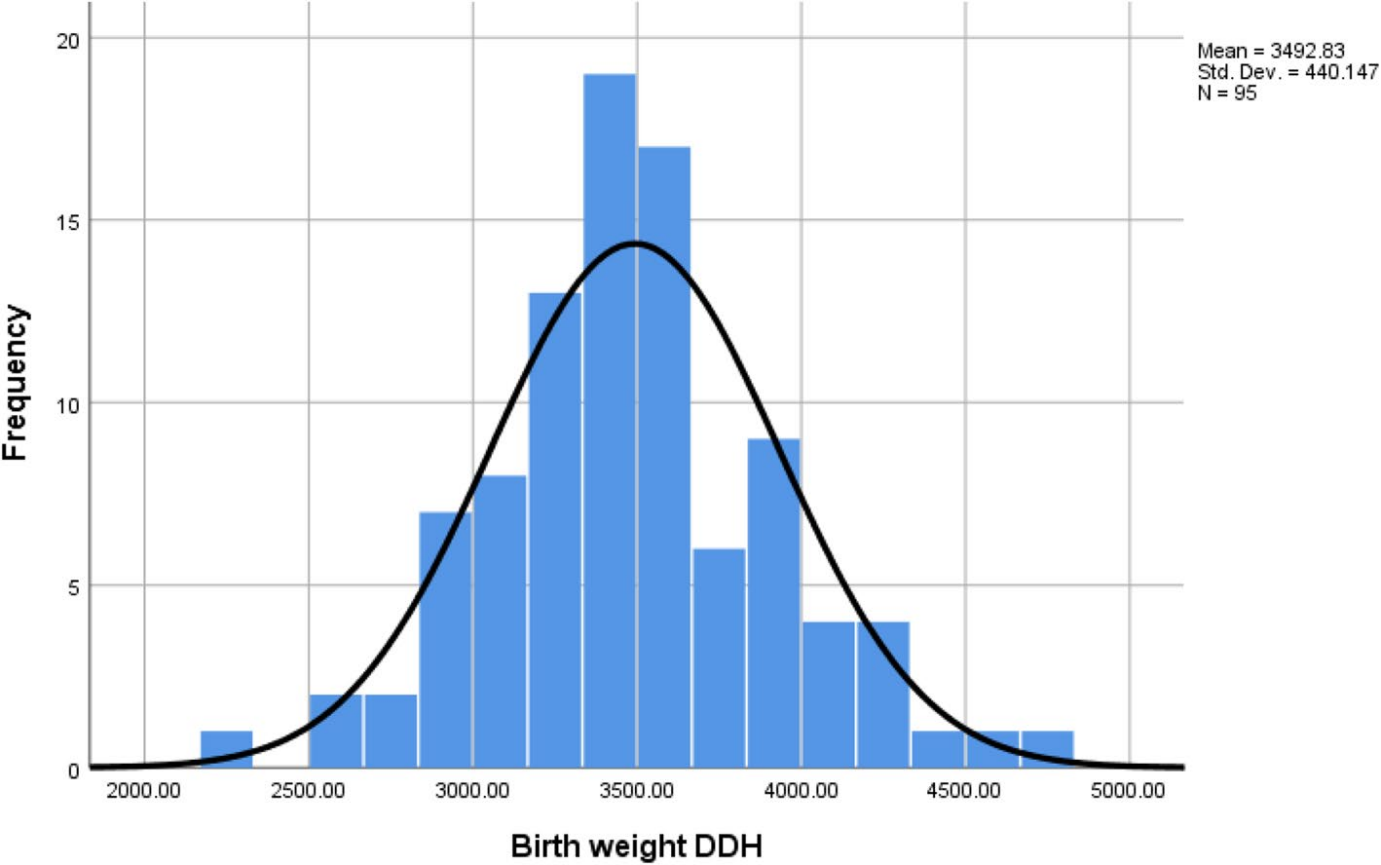
Histogram showing DDH birth weight frequency, with a normal distribution line overlaid. This data shows a normal skew

**Fig. 4:**
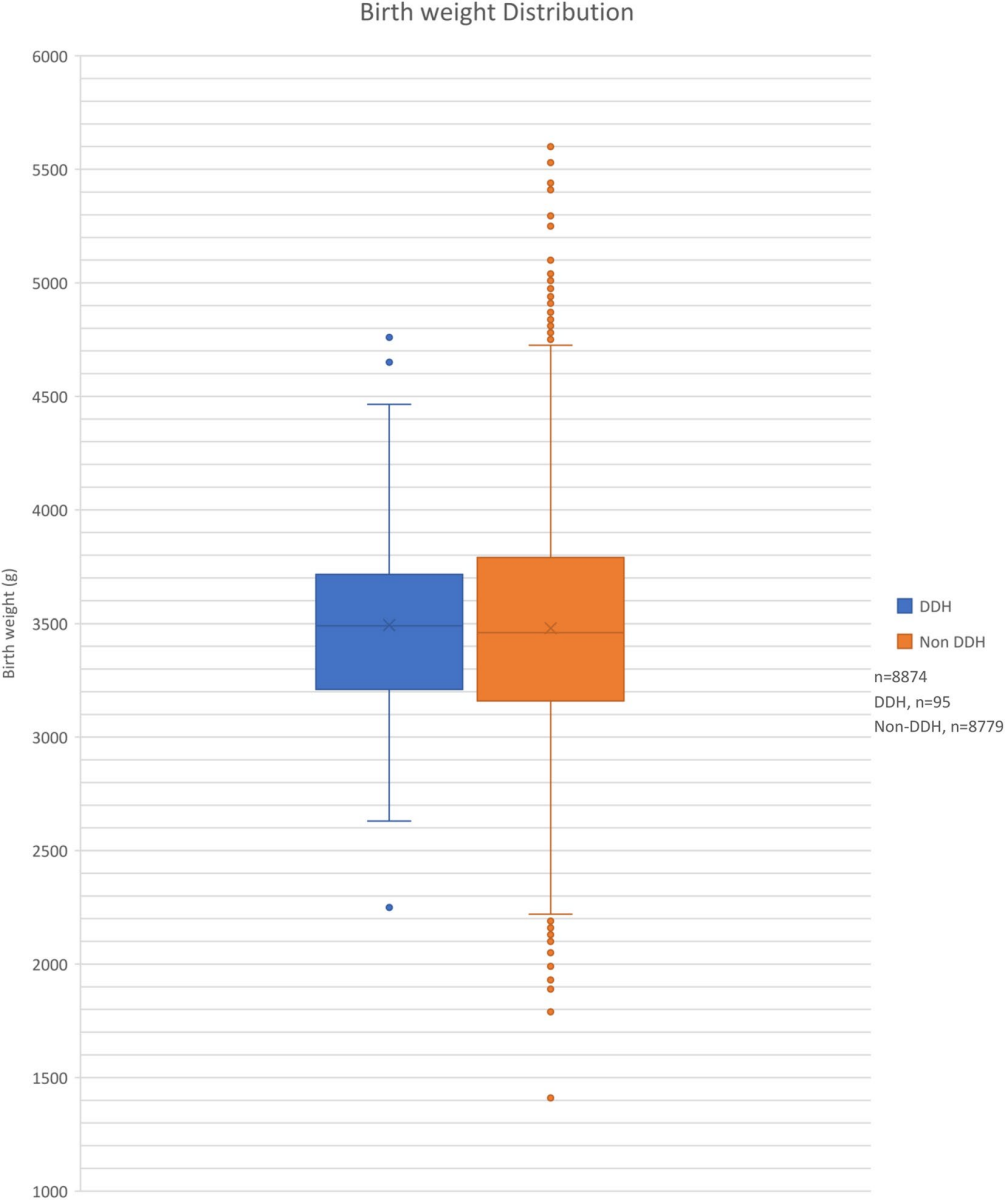
Boxplot comparing the birth weight of babies born with and without DDH. Means, minimum, maximum and interquartile ranges of the data sets are displayed

Both data sets were normally distributed (Shapiro Wilk Test (*p* = 0.308 Non DDH and *p* = 0.629 DDH), with a normal distribution being a significance of *p* = 0.05 or greater). Mean birth weight of the non-DDH group was 3479.8 g (SD 471.9 g) and of the DDH group was 3492.8 g (SD 440.1 g) (Figs. 2 and 3, respectively). There was no statistically significant difference between the two groups (Independent *t* test, *p* = 0.789) (Fig. 4).

Male patients represented 51.7% (*n* = 4592), and female patients 48.2% (*n* = 4281) of the population, with 0.01% (*n* = 1) classified as not known. Within the DDH data group, 86.3% of patients were female (*n* = 82) and 13.7% of patients were male (*n* = 13), in keeping with the established gender disparity of DDH [3].

The mean birth weight of males in this cohort was 3416.6 g and of females was 3293.1 g. This was statistically significantly different (*p* < 0.001). Sub analysis of birth weights of females with DDH (mean 3498.1 g (SD 427.6 g)) and without DDH (mean 3406.7 g (SD 449.9 g)) showed no significant difference (*p* = 0.68). The same was found in males with DDH (mean 3459.4 g (SD 531.1 g)) and without DDH (mean 3546.8 g (SD 481.7 g)), (*p* = 0.513).

**Fig. 5:**
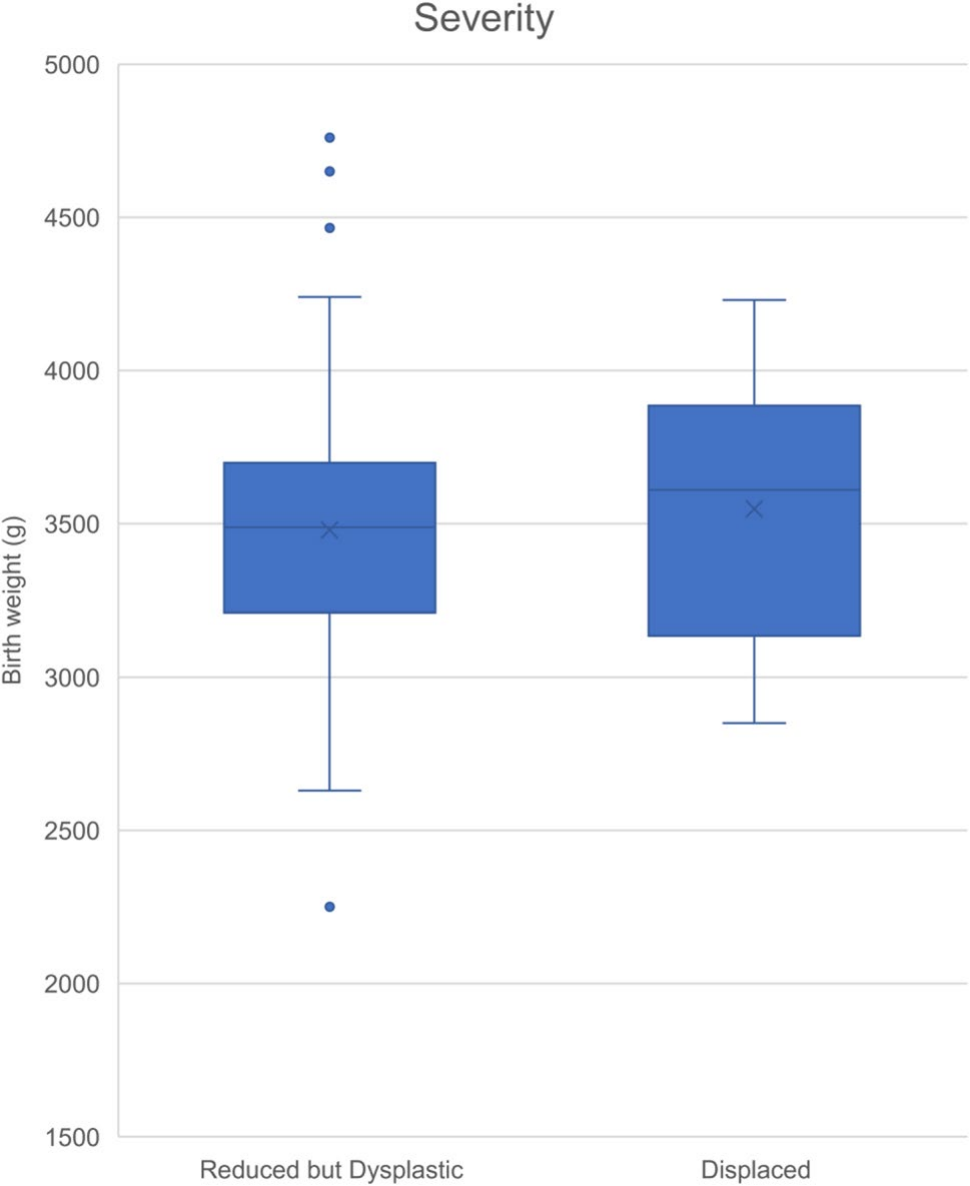
Box and whisker plot comparing the means, minimum and maximum and interquartile ranges of the two types of severity analysed; Reduced but Dysplastic vs Displaced

Birth weights of infants with displaced hips (dislocated/subluxed, cf Graf 3 or 4) were compared to those infants with Reduced but Dysplastic hips (cf Graf 2b, c and d). Within the DDH cohort, 80% (*n* = 76) had the less severe ‘Reduced but dysplastic’ form of DDH, and 20% (*n* = 19) had the more severe ‘Displaced’ form of DDH. Mean birth weights of the groups were 3479.0 g (SD 446.5) and 3458.2 g (SD 420.6 g) respectively, with no statistical difference found between the two (*p* = 0.543, Independent *t* test) (Fig. 5). The gender discrepancy was consistent across all DDH subgroups of severity, namely 86% of all DDH hips, 86% of reduced/dysplastic hips and 89% of displaced hips were female.

With progressive growth in utero, longer gestation babies are expected to be heavier. This was seen in this cohort, with mean birth weight of those babies born at 38–40 weeks (3297.9 g) being less than those born at 40–42 weeks (3581.6 g) (*p* < 0.00001). The difference in birth weight of DDH babies born within these timeframes was also significant (3291.2 g versus 3621.5 g), with the later born babies being heavier, as expected (*p* = 0.0003). However, despite the heavier birth weights of the longer gestation term babies, DDH incidence did not increase past 40 weeks birth. The incidence of DDH in babies born 38–40 weeks was 1.15%, versus 1.04% in those born 40–42 weeks (*p* = 0.64).

Breech presentation is an established risk factor for DDH, so was sub-analysed as a potential confounding factor. Of the 95 babies with DDH, 27 (28.4%) were breech. This is compared to 364 (4.1%) in the wider population. The breech babies with and without DDH had similar birth weights (mean 3344.1 g and 3361.6 g respectively, *p* = 0.8). The nonbreech babies with and without DDH had similar birth weights (3551.9 g and 3483.4 g respectively, *p* = 0.23). Breech babies in this cohort had lower birth weights than the babies who were vertex presentation (*p* < 0.0001), yet have a higher incidence of DDH.

## Discussion

This cohort study is novel with respect to the primary aim being solely the association of birth weight and DDH. Birth weights of babies in our study with and without DDH are entirely matched, both overall and within all sub-group analyses. This study has failed to demonstrate any link or association between DDH and birth weight, plus it casts doubt on DDH being related to any ‘packaging problem’.

With regard to the gender discrepancy of DDH, it is worth considering that females are born on average 120 g lighter than their male counterparts, but being female is considered a risk factor for DDH [3]. This is unlikely to be related to them having lower birth weight than males as sub analysis showed no difference of DDH prevalence between lower and higher birth weight females, nor lower and higher birth weight males. It, therefore, seems more likely that females are at a higher risk of DDH for a different reason than birth weight.

An important sub analysis was for the severity of DDH, to evaluate whether birth weight could have an effect on causing hip displacement, not just isolated acetabular dysplasia. Interestingly, the cohort of displaced or dislocated hips (*n* = 19, cf Graf 3 or 4 hips) had lower birth weight than those with isolated acetabular dysplasia and those babies without DDH.

Further sub-analysis of the later gestation babies, which had higher birth weight as expected, revealed no higher incidence of DDH. Were DDH to be caused by tight packaging in utero as some studies have suggested [5, 9, 10, 27–31] then the DDH incidence should climb in longer gestation babies. This was not the case, which further undermines the belief that birth weight, or even packaging disorders, contribute to the development of DDH.

Sub-analysis on breech presentation showed, yet again, no link between DDH and birth weight. The birth weights of babies with and without DDH, when sub-analysed according to being breech or not, were equal.

It was interesting to note that breech babies in this population demonstrated lower birth weights than non-breech babies (*p* < 0.0001). This observation has been reported before [33], but the reason for this is unclear. It may be due to planned Caesarian sections for breech being performed at slightly earlier gestational dates, or before babies became macrosomic. Breech babies are at risk of DDH, as demonstrated once again in this population, but this cannot be attributed to higher birth weight as birth weights were, in fact, lower in breech babies.

This study has numerous strengths. It was deliberately kept within one institution to minimise any known or unknown confounding factors. Whilst more than 95 infants are treated at our institution each year for DDH, many are from a wider geographical area than those solely born at our institution. These patients could have been included to increase the numbers of babies with DDH in this study. However, we did not have access to data on the remaining population from each respective referral region. Future work could repeat this study at a national level using clinical coding.

Of all of the patients included in this study only 0.2% (*n* = 18) were excluded due to missing data. Of the DDH group 1.1% (*n* = 1) were excluded, and of the non-DDH group 0.19% (*n* = 17) were excluded. The HSCIC Hospital Episode Statistics reportedly have information lacking with regard to the status of over 10% of all UK births [34]. Thus the limitations of big data were not present in this study. Furthermore, this dataset is representative of the national population, as confirmed via numerous external validations. The birth weights within this study matched the reported national values [26] and the gender distribution of babies with DDH was in keeping with established ratios [4].

Late detected cases were included in this study, but it is possible that there are more babies born at our institution within the study timeframe who are yet to be diagnosed. These infants would now be greater than two years old, so numbers would be small [32], and should not alter the conclusions of this study. It is also worth noting that some people do not present with DDH until they are in adulthood. They therefore too will have been missed in this study.

In this study White British was the most heavily represented ethnicity. Sub analysis according to ethnicity did not reveal any notable differences, but numbers of different ethnic groups were small and analysis was limited. Some of the sub-analyses performed had sub-group sizes smaller than the power calculation. As such, there could feasibly be Type 2 error here.

Specific factors not investigated in this study include head circumference, method of delivery and liquor volume. Head circumference could be considered relevant, in the theory of packaging aetiology. This data was not specifically analysed, but head circumference is known to be proportional to birth weight in term babies [35]. As only term babies were included in this study, then a normal range of head circumference can be expected. Mode of birth has not been independently linked to DDH [15] and was not investigated. Breech babies in the UK are delivered by planned C-section, as such mode of delivery is heavily linked to breech presentation, which was analysed. Data on liquor volume was not available, thus the effect of oligohydramnios could not be considered.

Whilst this study has shown no link between birth weight and DDH, it has not addressed the possible link to birth weight and progression of hip immaturity. Many babies screened by USS at around 6 weeks of age have borderline hips (cf Graf 2a) who undergo re-scan a few weeks later. Most of these develop normally and get discharged, however, some remain dysplastic and go on to have treatment after serial re-scans (cf Graf 2b). It would be interesting to consider whether birth weight has an impact on whether borderline hips at 6 weeks of age become normal or remain dysplastic.

To conclude, the findings of this study clearly dispute any suggestion that DDH is linked to birth weight. As such, birth weight should not be considered as an independent risk factor for screening programmes. Furthermore, they challenge the theory that DDH is a packaging disorder. If DDH was due to a packaging effect, it would manifest as a higher incidence in higher birth weight babies. Furthermore, higher birth weight would theoretically cause more severe forms of DDH due to more restricted uterine space. It can now be concluded that birth weight is entirely irrelevant in the aetiology of DDH. With no link to birth weight, the likelihood of DDH being a packaging disorder is low.
